# Semi-Mechanistic Pharmacokinetic Model to Guide the Dose Selection of Nimotuzumab in Patients with Autosomal Dominant Polycystic Kidney Disease

**DOI:** 10.3390/pharmaceutics12121147

**Published:** 2020-11-26

**Authors:** Niurys de Castro-Suárez, Mirjam N. Trame, Mayra Ramos-Suzarte, José M. Dávalos, Raymed A. Bacallao-Mendez, Anaelys R. Maceo-Sinabele, Víctor Mangas-Sanjuán, Gledys Reynaldo-Fernández, Leyanis Rodríguez-Vera

**Affiliations:** 1 Pharmacy Department, Institute of Food and Pharmacy, University of Havana, Havana 11300, Cuba; niurys@ifal.uh.cu (N.d.C.-S.); gledysrf@ifal.uh.cu (G.R.-F.); leyanis@ifal.uh.cu (L.R.-V.); 2 AVROBIO Inc., Department of Translational Data Sciences and Advanced Analytics, Cambridge, MA 02139, USA; mirjam.trame@avrobio.com; 3 Center of Molecular Immunology (CIM), Havana 11300, Cuba; mayra@cim.sld.cu; 4 National Institute of Nephrology (INEF), Havana 10400, Cuba; jdavalos@infomed.sld.cu (J.M.D.); raymed@infomed.sld.cu (R.A.B.-M.); 5 National Center Coordinating of Clinical Trials (CENCEC), Havana 11300, Cuba; anaelys@cencec.sld.cu; 6 Department of Pharmacy and Pharmaceutical Technology and Parasitology, University of Valencia, 46100 Valencia, Spain; 7 Interuniversity Research Institute for Molecular Recognition and Technological Development, Polytechnic University of Valencia, University of Valencia, 46100 Valencia, Spain

**Keywords:** nimotuzumab, EGFR, autosomal dominant polycystic kidney disease, semi-mechanistic pharmacokinetics, population analysis, NONMEM

## Abstract

Autosomal dominant polycystic kidney disease (ADPKD) is a genetic disease characterized by an overexpression of epidermal growth factor receptor (EGFR). Nimotuzumab is a recombinant humanized monoclonal antibody against human EGFR. The aim of this study was to develop a population pharmacokinetic model for nimotuzumab and to identify demographic and clinical predictive factors of the pharmacokinetic variability. The population pharmacokinetics (PopPK) of nimotuzumab was characterized using a nonlinear mixed-effect modeling approach with NONMEM^®^. A total of 422 log-transformed concentration-versus-time datapoints from 20 patients enrolled in a single-center phase I clinical trial were used. Quasi steady state approximation of the full TMDD (target-mediated drug disposition) model with constant target concentration best described the concentration-time profiles. A turnover mediator was included which stimulates the non-specific clearance of mAb in the central compartment in order to explain the reduced levels at higher doses. Covariates had no influence on the PK (pharmacokinetics) parameters. The model was able to detect that the maximum effective dose in ADPKD subjects is 100 mg. The developed PopPK model may be used to guide the dose selection for nimotuzumab during routine clinical practice in patients with polycystic kidney disease. The model will further support the ongoing investigations of the PK/PD relationships of nimotuzumab to improve its therapeutic use in other disease areas.

## 1. Introduction

Autosomal dominant polycystic kidney disease (ADPKD) represents the most prevalent (1/1000–1/2500 live births) genetic disease affecting the kidney, resulting in a massive cystic enlargement of renal tubules caused by an enhanced epithelial cell proliferation, altered fluid secretion, changes in the extracellular matrix, extrarenal alterations, and mispolarization of several membrane proteins such as the epidermal growth factor receptor (EGFR) leading to cyst formation in both kidneys [1,2]. Renal insufficiency is, therefore, caused by an expansion of cysts which compress viable renal tissue. Several studies suggest that EGFR, ErbB2 and their ligands enhance the proliferation of tubular epithelial cell, leading to cyst formation. The over-expression of EGFR and its mislocation to the apical membrane allows its binding to EGF, activating the signaling cascade and, ultimately, the cell proliferation [1,3]. Current drug therapy is focused on the treatment of symptoms and complications, such as hypertension. Several pathophysiological targets (signaling molecules and pathways) have been recognized in the transition from normal tubular epithelial to the cystic phenotype [4]. Tolvaptan (vasopressin V2 receptor antagonist) is the first treatment able to reduce the increase in total kidney volume and diminish the loss of renal function in adult ADPKD patients [5], but it is associated with occasional hepatic injury [5]. In this sense, tolvaptan is indicated in patients with high probability of developing end-stage renal failure [4,5].

The evidence from several studies demonstrates the significant role of the EGFR pathway in renal cystic epithelial proliferation. The inhibition of EGFR activity through genetic strategies or the use of tyrosine kinase inhibitors reduce renal cyst formation and enlargement in murine and human models of ADPKD [6,7]. Nimotuzumab (also known as TheraCIM^®^, Theraloc^®^, CIMAher^®^, BIOMAb-EGFR^®^, Tai Xin Sheng^®^, OSAG-101 or YMB-1000) is a humanized monoclonal antibody (mAb), developed at the Center of Molecular Immunology (Havana, Cuba). It is a genetically engineered IgG1 with intermediate affinity (K_d_ = 2.1 × 10^−8^ M) to EGFR, has a molecular weight of 150 KDa and exhibits a G0 glycosylation pattern [8]. Nimotuzumab was derived by transplanting the CDRs of the murine mAb ior egf/r3 to human framework (Eu for heavy chain and REI for light chain). This mAb interacts with the extracellular region of EGFR (domain III) [9], leading toa potent antiproliferative, antiangiogenic and pro-apoptotic activities in overexpressed EGFR epithelial tumors. [10,11]. The pharmacokinetics (PK) of nimotuzumab after single dose and multiple dose administrations has been characterized either by noncompartmental [12,13] or classical compartmental analyses [10]. However, the empirical description of the nonlinear kinetic processes provided by these approaches is insufficient to characterize the mAb-target interactions [14]. Therapeutic mAbs such as nimotuzumab able to interact with receptors located on the cell surface usually show nonlinear PK properties due to target-mediated drug disposition (TMDD) [15]. A TMDD model is a nonlinear system of differential equations that describes the dynamic system of antibodies binding to the target molecule [15,16]. An adequate development of the TMDD model requires to have detailed drug concentration, target concentration and/or the drug-target complex concentration data. However, both the target pharmacology and the target dynamics are not always experimentally available or, if available, it becomes challeging to characterize the initial binding process, leading to identifiability issues. Therefore, simpler models based on approximations, such as those assuming rapid binding quasi equilibrium (QE) models, quasi-steady-state models (QSS) and Michaelis–Menten models, have been developed to enable fitting the model to data more feasibly [17]. The first semi-mechanistic TMDD model for nimotuzumab after repeated intravenous administration in breast cancer patients was proposed in 2015 [18].

The objectives of this study were (1) to develop a target-mediated disposition model for nimotuzumab in adult patients with ADPKD; and (2) to select the dosing strategy of nimotuzumab able to optimize nimotuzumab’s pharmacokinetic properties.

## 2. Materials and Methods

### 2.1. Study Design and Patient Eligibility

Twenty patients with ADPKD were enrolled in a single-center, open-label, dose-escalation phase I clinical trial. A single intravenous 30 min infusion of nimotuzumab was administered in four cohorts (five patients per cohort) of fixed dose levels (50, 100, 200 and 400 mg) of nimotuzumab. The product is formulated as a colorless and clear solution, without insoluble matters. Each vial contains nimotuzumab, dibasic sodium phosphate (Na2HPO4), monobasic sodium phosphate (NaH2PO4), sodium chloride, polysorbate 80 and water for injection. Blood samples were collected before the first dosing and at 0.5, 1, 2, 6, 12, 24, 36, 48, 72, 96, 120, 144, 168, 240, 338, 432, 504, 552, 576, 600, 624, 648 and 672 h post-administration. Serum was collected from experimental samples and then stored at −20 °C. A receptor-binding ELISA (enzyme-linked immunosorbent assay) was used to quantify the serum concentrations of nimotuzumab, using the antigen HER 1, recombinant extracellular of EGFR domain to capture nimotuzumab from serum samples [13]. Sheep antihuman IgG gamma chain specific alkaline phosphate (Sigma Chemical, A-3188, St. Louis, MO, USA) was selected to quantify the bound nimotuzumab and para-nitro-phenyl-phosphate diluted in diethanolamine was used to quantify serum nimotuzumab. Absorbance detection was established at405 nm and the lower limit of quantification of nimotuzumab in human serum was 7.8 ng/mL.

The inclusion criteria were patients with ADPKD diagnosis with good performance status, normal hematological conditions, normal hepatic function, as well as a Glomerular Filtration Rate ≥ 50 mL/min/1.73 m^2^. Patients with urinary protein excretion higher than 1 g in 24 h were excluded from study participation. Other exclusion criteria included previous treatments with murine anti-EGFR antibodies, pregnancy or lactation, serious chronic diseases and active infections. The Cuban clinical trial was authorized by the National Regulatory Agency (CECMED) on 7 March 2009 (number 442/05.014.08-B, volume 06, folio 000402). The study was conducted in accordance with the principles of the Declaration of Helsinki and Good Clinical Practice guidelines and was approved in 2009 by the Ethics Review Committee for human subject’s protection in clinical trials at the National Institute of Nephrology. All patients signed a written consent form before their inclusion in the clinical trial.

### 2.2. Data Analysis

Experimental serum observations of nimotuzumab were logarithmically transformed. A non-linear mixed effects modelling approach was implemented with the software NONMEM^®^ (v7.4, ICON plc Development Solutions, Hanover, MD, USA). Stochastic Approximation of the Expectation Maximization and the Importance Sampling Estimation method were considered for parameter estimation.

An exponential model was selected to account for the inter-individual variability (IIV), which was associated with the typical population PK parameters in order to avoid negative individual estimates. An additive model on the logarithmic scale was implemented to characterize the residual unexplained variability (RUV). During model development, non-diagonal elements of the Ω variance-covariance matrix were assessed. Model selection was based on the statistical decrease (7.879 units for 1 degree of freedom) of the objective function value provided by NONMEM^®^ and approximately equal to -2×log(likelihood) (-2LL) for nested models together with the visual inspection of the goodness-of-fit (GOF) plots. The Akaike information criteria (AIC) were considered for the selection of non-nested models.

Model evaluation was performed through prediction-corrected visual predictive checks (pc-VPC) [19]. Briefly, one thousand simulated datasets were simulated and the 2.5th, 50th and 97.5th percentiles for every simulated study and sampling time period were calculated. Then, the 95% prediction intervals of the above described percentiles were calculated and displayed graphically together with corresponding percentiles computed from raw data. The nonparametric bootstrap method with replacement was used to assess the stability and precision of parameter estimates of the final model. Five hundred simulations were conducted by resampling from the original data. The final population PK model was fitted to each data set and parameter estimates were obtained. replicate data sets and parameter estimates for each of the replicate data sets were obtained. The median values and the 95% confidence intervals of the parameters obtained were compared with those estimated from the original data.

Software R (http://cran.r-project.org, version 3.5.0) was considered for graphical and statistical analysis. Pc-VPC and bootstrap analyses were performed using PsN [20].

### 2.3. Population Pharmacokinetic Analysis

**Figure 1:**
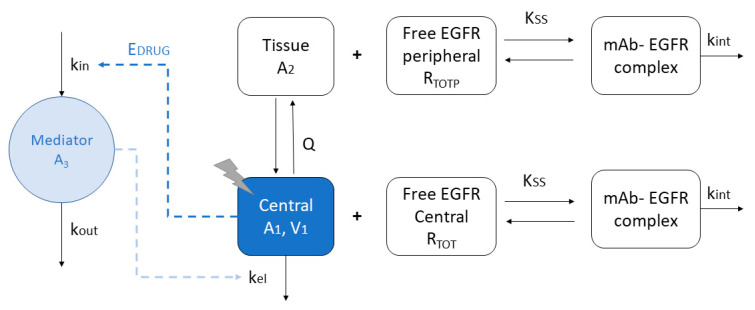
Schematic representation of the quasi steady state approximation (QSS) with constant number of total receptors of the TMDD (target-mediated drug disposition) model, which explicitly includes mAb-target binding in central and peripheral tissue compartments, receptor turnover and internalization of the mAb-target complex from the central compartment. K_SS_, steady-state rate constant; Q, inter-compartmental clearance; A_2_, amount of the mAb in the tissue compartment; A_1_, amount of free mAb in the serum compartment; V_1_, apparent volume of distribution of the central compartment; R_TOT_, concentration of free target in central compartment; R_TOTP_, concentration of free target in tissue compartment; k_out_, rate constant for the first-order degradation of the mediator (A_3_). A_3_ increases nimotuzumab’s clearance (CL).

A QSS TMDD model was able to characterize the time-course of nimotuzumab in ADPKD patients. A representation of the population PK model is depicted in Figure 1.

Following intravenous infusion, nimotuzumab is distributed into a central compartment with an apparent volume of distribution (V_1_) and further distributed to a peripheral compartment (Q) with an apparent volume of distribution (V_2_). The elimination of free nimotuzumab was characterized by a non-specific (linear) clearance (CL) process or by binding to its target (i.e., EGFR), which assumed a first-order process (k_int_). No elimination process of the complex was assumed from the peripheral compartment [21]. The enhanced CL of nimotuzumab observed at higher doses was described using a mediator (A_3_), whose synthesis (k_in_) is activated due to free concentrations of nimotuzumab (A_1_) through a sigmoid function (S_max_, S_50_ and γ). A reversible binding of nimotuzumab with EFGR was assumed, parameterized as K_ss_ = (k_off_ + k_int_)/k_on_, where k_off_ and k_on_ represent the first-order rate constant involved in the dissociation and formation of the nimotuzumab-EGFR complex, respectively. A turn-over model was implemented to describe the time-course of EGFR, assuming a zero-order synthesis process (k_syn_) and a first-order degradation process (k_deg_). Steady-state conditions of EGFR were considered before nimotuzumab administration (k_syn_ = k_deg_ × R_0_, where R_0_ is the baseline concentration of free EGFR). Different target concentrations were considered for the central (R_tot_) and peripheral (R_totp_) compartments. Total concentration of nimotuzumab was expressed as the sum of the free nimotuzumab concentration (A_1_/V_1_) and nimotuzumab-EGFR complex (R_tot_*A_1_/V_1_/(K_ss_ + A_1_/V_1_)). The differential equations of the QSS TMDD model are shown as follows [21,22]:

(1) $$\frac{dA_{1}}{dt}=\frac{-\left(\frac{CL\;\cdot \;A_{3}}{V_{1}}\;+\;\frac{Q}{V_{1}}\right)\cdot \;A_{1\;}+\;\frac{Q}{V_{2}}\cdot \;A_{2\;}-\;\frac{k_{int\;}\cdot \;R_{tot\;}\cdot \;A_{1}}{\left(K_{ss}\;+\;\frac{A_{1}}{V_{1}}\right)}}{1\;+\;\frac{R_{tot\;}\cdot \;K_{ss}}{{\left(K_{ss}\;+\;\frac{A_{1}}{V_{1}}\right)}^{2}}}$$

where *A*_1_ represents the nimotuzumab free amount in the central compartment, *A*_2_ is the free nimotuzumab amount in the peripheral compartment, *V*_1_ and *V*_2_ are the apparent volume of distribution of the central and peripheral compartment, respectively, CL represents the non-specific clearance, *Q* is the distribution clearance of free nimotuzumab between the central and peripheral compartment, *R*_tot_ is the EGFR in the central compartment, *k*_int_ represents the internalization rate for nimotuzumab-EGFR and *K*_ss_ is the *Q*_SS_ constant for the EGFR.

(2) $$\frac{dA_{2}}{dt}=\frac{\frac{Q}{V_{1}}\cdot A_{1-\;}\frac{Q}{V_{2}}\cdot A_{2}}{1\;+\frac{R_{totp\;}\cdot K_{ss}}{{\left(K_{ss}\;+\frac{A_{2}}{V_{2}}\right)}^{2}}}$$

where *R*_totp_ is the EGFR in the peripheral compartment.

(3) $$\frac{dA_{3}}{dt}=k_{in}\cdot \left(1+\;\frac{S_{max}\cdot \;A_{1}{}^{\gamma }}{\left(S_{50}{}^{\gamma }+\;A_{1}{}^{\gamma }\right)}\right)-\;k_{out}\cdot \;A_{3}$$

where *k*_in_ represents the zero-order synthesis rate constant, *k*_out_ the first-order elimination rate constant, *A*_3_ is the amount of mediator, *S*_max_ is the maximal effect of the stimulation, *S*_50_ is the concentration of free nimotuzumab in the central compartment that achieves the half of *S*_max_ and γ is the Hill coefficient of the sigmoid function [16].

Initial conditions for Equations (1)–(3) are the following:

A_1_(0) = 0; *A*_2_(0) = 0; *A*_3_(0) = 1;
R_tot_(0) = *R*_0_ = *k*_syn_/*k*_deg_
*k*_in_ = *k*_out_ × *A*_3_(0)

Covariates investigated for their influence on PK parameters included continuous characteristics as demographics (body weight, height, age, body surface area), laboratory values (creatinine clearance (CrCL), serum creatinine) and others (total kidney volume (TKV), total cyst volume (TCV)), as well as the categorical characteristics (sex, race). Graphical and numerical analyses were used to assess the relationship between patient covariates and individual PK parameters only when eta-shrinkage was less than 35% [23]. A stepwise covariate model-building (SCM) in an univariate manner [20,24] allowed to evaluate the statistical significance (0.05 and 0.01 for the forward addition and backward elimination, respectively) of the parameter-covariate relationship.

### 2.4. Simulations

A simulation-based analysis was conducted using the typical population PK parameters to predict the time-course of nimotuzumab and mediator in humans. A deterministic simulation was performed assuming a single IV infusion administration of nimotuzumab at different dose levels (50, 100, 200, 400, 800 and 1200 mg) in a patient with ADPKD. Concentration-time profiles of nimotuzumab (central and peripheral compartment) and mediator were generated up to 1000 h (~16.6 days).

## 3. Results

### 3.1. Patients and Data Collection

A total of 422 serum nimotuzumab concentrations from 20 patients were used for the population PK analysis. None of the observations were below the limit of quantification. Table 1 summarizes the main demographic and anthropometric characteristics of the studied population. The patient population was predominantly Caucasian (75%) and female (70%). At the start of treatment, the median age was 42 years and body weight 65.7 kg. At baseline, the mean total kidney volumes (TKV) (women 924.14 ± 404.27 mL; men 822.18 ± 486.22 mL) were consistently larger than those reported for adult normal kidneys [25].

The adverse effects observed for nimotuzumab [1] were lower than reported for other anti-EGFR antibodies, e.g., cetuximab [26], which has been attributed to its intermediate affinity. The dermatological toxicity (rash and acne) was the principal adverse effect related to nimotuzumab administration. It is a common effect of anti-EGFR mAbs.

### 3.2. Population Pharmacokinetic Analysis

The developed model adequately describes the observed PK of nimotuzumab in ADPKD subjects. The concentration-time profiles were best described by a 2-compartment model with concentration-dependent protein binding in the central and the peripheral compartment. The addition of a turnover mediator which stimulates the non-specific CL of nimotuzumab statistically improved the model fit (*p* < 0.01).

**Figure 2:**
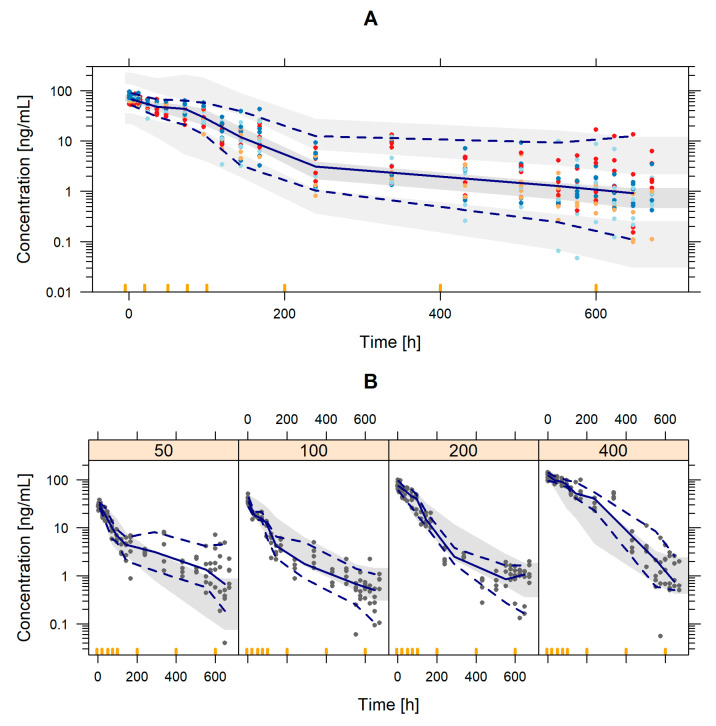
(**A**) Prediction-corrected visual predictive check of the final population PK model. Shaded areas represent the 95% prediction intervals of the 2.5th, 50th and 97.5th percentiles of the simulated data. Red, orange, light blue and blue circles represent nimotuzumab observations corresponding to the dose level of 50, 100, 200 and 400 mg. Lines represent the 2.5th, 50th and 97.5th percentiles of the raw data. (**B**) Prediction-corrected visual predictive check of the final population PK model. Shaded areas represent the 95% prediction interval of the 50th percentile of the simulated data. Grey dots represent nimotuzumab observations and lines represent the 2.5th, 50th and 97.5th percentiles of the raw data.

**Figure 3:**
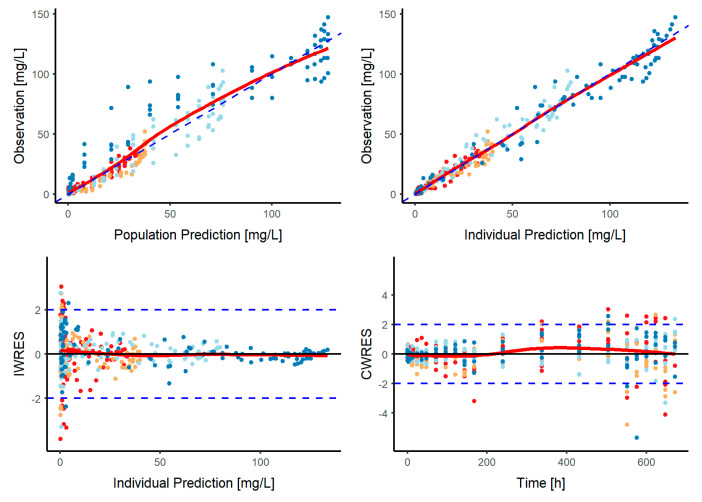
Model performance of the final population pharmacokinetic model. Goodness of fit plots. Red, orange, light blue and blue circles represent nimotuzumab observations corresponding to the dose level of 50, 100, 200 and 400 mg. The red solid line represents the non-linear regression and the blue dotted line represents the line of identity. IWRES: individual weighted residuals, CWRESI: conditional weighted residuals.

The adequacy of the model to capture the nimotuzumab time-course profiles is depicted in Figure 2, which shows the pc-VPC of all data and is stratified by dose level, the latter showing a slight model-misspecification possibly due to the small number of patients in each group and the slight bias in characterizing the effect of the mediator at extreme doses. Standard GOF plots are shown in Figure 3.

None of the 95% CI included the null value and the final parameter estimates lied within the 95% CI obtained from the bootstrap results. Parameter precision, measured as RSE, was calculated from the standard error (SE) and median value results from the bootstrap analysis. The parameter estimates of the base model are displayed in Table 2. 

Clearance of nimotuzumab from the central compartment was faster in patients with ADPKD (9.64 × 10^−3^ L/h) compared to a previous population of patients with advanced breast cancer (7.03 × 10^−3^ L/h). Total distribution volume at steady state (V_ss_) was 2.64 L; this value was smaller compared with the aforementioned study in oncological patients [18]. The steady-state constant K_ss_ (15.5 mg/L) is in the range of the reported values for therapeutic monoclonal antibodies [22]. The internalization rate constant (k_int_) was slightly higher than the elimination rate constant of the free drug from the central compartment (CL/V_1_).

The apparent amount of EGFR in the peripheral compartment was significantly higherthan in the central compartment, according to the value of R_totp_ (956 mg/L) and R_tot_ (1.05 × 10^−2^ mg/L). This suggests that the capacity of EGFR inhibition at the cyst level (peripheral compartment) may be much higher than observed in central compartment, and the peripheral compartment is responsible for most of the nonlinearity seen in nimotuzumab PK. However, the estimates for R_tot_ and R_totp_ as apparent amounts of available EGFR might not represent the actual amounts of receptor in both compartments, as amounts of EGFR were not experimentally available.

IIV was evaluated in several PK parameters but it was statistically significant on R_totp_ (135%) and K_out_ (197%). A residual error value of 48% was obtained. None of the covariates were statistically significant regarding PK parameters to partially explain the high inter-individual random effects. Accordingly, the early-developed base model was selected as the final one.

### 3.3. Simulations

**Figure 4:**
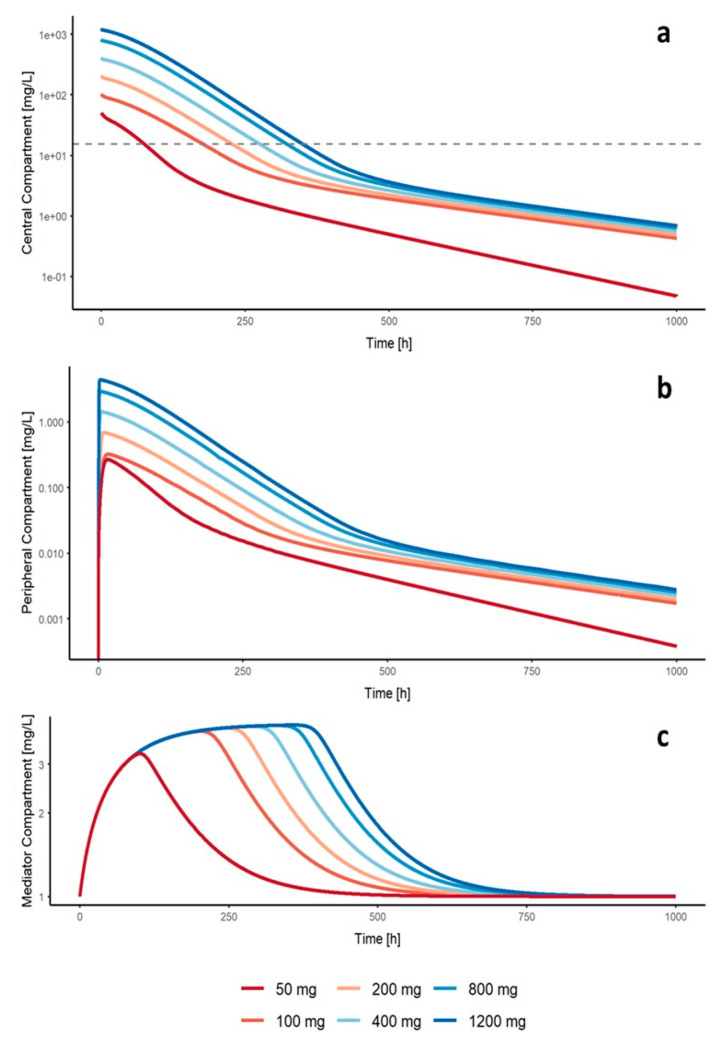
Predicted concentrations from the final model. Predicted concentrations from the start of the treatment assuming a single administration of 50, 100, 200, 400, 800 or 1200 mg of nimotuzumab in a patient with autosomal dominant polycystic kidney disease in the central (**a**), peripheral (**b**) and mediator compartments (**c**).

Figure 4 illustrates the typical PK profiles of nimotuzumab assuming a single administration of 50, 100, 200, 400, 800 or 1200 mg of nimotuzumab in a patient with ADPKD in the central, peripheral and mediator compartments. The dose levels selected (50–1200 mg) covered experimentally (50, 100, 200, 400) and non-experimentally (800, 1200 mg) available dose levels in order to predict the impact of higher dosing schedules on the PK properties of nimotuzumab. The influence of mediator concentrations enhances the CL of nimotuzumab in the central compartment, since the ratio between nimotuzumab and mediator concentrations is inversely related to the dose level. The effect of nimotuzumab to activate the generation of the mediator is increased among all dose levels evaluated, reaching the maximum effect with a dose of 100 mg (S_50_ = 8.57 mg/L). The duration of mediator activation is directly related to the concentration of the mAb in serum, which remains above the S_50_ for a longer period of time at higher doses. This phenomenon favors a faster elimination in the early stages of the kinetic profile (<125 h) of the nimotuzumab concentrations in the central compartment at a dose of 50 mg compared to the other dose levels evaluated.

## 4. Discussion

A TMDD model with two saturable binding processes (central and peripheral) was able to characterize the non-linear PK properties of nimotuzumab in ADPKD patients in a single dose regimen. The binding of nimotuzumab to EGFR in the renal cysts was implemented in the peripheral compartment, since EGFR is localized at the basolateral membrane in renal tubular cells and at both apical and basolateral membranes of cyst epithelial cells [1,6,27,28]. The binding affinity of nimotuzumab to EGFR in central and peripheral compartments is identical. Furthermore, the model incorporates a turnover mediator which stimulates the non-specific CL of nimotuzumab. Our study, for the first time, describes the PK behavior of nimotuzumab in a non-oncological disease.

Several approaches for TMDD have been developed [15,16,17] in the last decade to explain how the PK of the mAb is affected by its binding to the pharmacological target. Those models assumed that the binding of the drug only occurs in the central compartment. However, other authors have developed TMDD models to represent binding occurring in both the peripheral tissue and central compartments. The model developed by Lowe et al. [29] is based on a QE assumption to model the behavior of any antisoluble ligand antibody. This model assumed that the elimination rate of the complex was the same for both compartments. Retlich et al. [30] developed a similar model to predict the behavior of the drug linagliptin, a dipeptidyl peptidase-4 (DPP-4) inhibitor. Retlich et al. [30] only considered differential equations for the amounts of the drug in the two compartments and the depot. The population PK model assumed that the binding affinities of linagliptin to soluble plasma DPP-4 and membrane-bound tissue DPP-4 are identical. The model proposed by Landersdorfer et al. [31] adequately predicted the drug concentrations and effects of vildagliptin on DPP-4. The equations for binding of vildagliptin to DPP-4 were based on the Michaelis–Menten equation.

Based on the final population PK model, nimotuzumab distributes into a central compartment volume (V_1_) of 2.63 L for a median body weight of 65 kg, and an even smaller peripheral volume of distribution of 9.92 × 10^−3^ L, which suggested limited distribution outside the central compartment. The intercompartmental clearance (Q) also indicated a limited distribution, which was consistent with the behavior of endogenous IgG. Compared with other mAbs, in this study nimotuzumab has a much smaller V_2_ [32]. The estimated non-specific clearance for nimotuzumab is quite close to the clearance of endogenous IgG (0.21 L/day) [32]. The PK of other therapeutic mAb, such as panitumumab, an anti-EGFR [33], trastuzumab, which binds to HER2 [34], pertuzumab, which inhibits the formation of the HER2 heterodimer [35], infliximab, which binds to human tumor necrosis factor α (TNF-α) [36], and rituximab, which is directed against the B cell receptor CD20 [37], have been characterized by 2-compartment models with only non-specific clearance ranging from 0.21 to 0.40 L/d.

A decrease of the target-mediated clearance is expected as long the target is saturated and, therefore, the dose increases. Then, the role of the target-mediated clearance becomes insignificant and mAb is cleared through catabolism by the reticuloendothelial system (RES) [35,38]. One key mediator of this process is the FcRn (mAb-neonatal Fc receptor), which protects the internalized antibody from rapid intracellular catabolism [39]. Unlike previous pharmacokinetic reports of mAb [32,33,40,41], the population PK analysis showed that at the administered doses of nimotuzumab, the non-specific CL pathway was the dominant component of total CL. In order to explain this behavior, a mediator which stimulates the non-specific CL was included. Model-based simulations (Figure 4) revealed that nimotuzumab is able to reach the maximum effect on the mediator with a dose of 100 mg. Simulations also suggest that EGFR is saturated after a single dose of 100 mg. This is consistent with the results from a previous PK analysis [13], where the total CL reached a plateau at 100 mg. We speculate the mediator could be related to the activity of the matrix metalloproteinases (MMPs) and FcRn in the kidneys. Matrix metalloproteinases are a large family of proteinases that remodel extracellular matrix (ECM) components and cleave a number of cell surface receptors [42]. Several studies on polycystic kidney disease have revealed cystic kidneys tubules synthesize and secrete high levels of MMPs, especially MMP-2, MMP-1 and MMP-9 [43,44]. Data from numerous studies suggest a role for MMPs in a number of renal pathophysiologies [43,45,46,47]. A recent study carried out on proteinuric patients and mice [48] showed that the inhibition of MMP enzymatic activity reduced renal proximal endocytic receptors, such as FcRn, shedding into the urine of proteinuric animals and preserved cell surface expression of these receptors in the proximal tubule epithelial cells (PTEC). They also found the decreased expression of these endocytic receptors to be related to the increased urinary excretion of protein. These findings suggest that MMPs may be contributing to the cleavage and regulation of renal proximal endocytic receptors in the PTEC in proteinuric conditions. This hypothesis should be further investigated since neither information regarding levels of MMPs nor urinary protein excretion data were available in these patients. 

Estimation of K_ss_ varied among different therapeutic mAb with 150 kDa molecular weight, with K_ss_ values ranging from 0.15 mg/L to 15 mg/L (1–100 nM) [22]. The steady-state constant K_ss_ estimated for nimotuzumab is within this wide range (15.5 mg/L). The model predicted K_ss_ to be higher than the equilibrium dissociation rate constant (K_d_) determined in vitro by surface plasmon resonance (K_d_ = 2.1 × 10^−8^ mol/L, k_on_ = 5.2 × 10^4^ (s mol/L)^−1^, k_off_ = 1.1 × 10^−3^ s^−1^) [8]. It is expected that K_ss_ (K_ss_ = K_d_ + k_int_/k_on_) [17] derived from modeling in vivo data would be higher than K_d_ because the antibody receptor complex is internalized in vivo, unlike the in vitro conditions of surface plasmon resonance measurements. It has been observed in many cases that in vitro K_d_ of a mAb is often different from the in vivo K_d_ (or K_ss_) [18,49].

A model-informed dose selection analysis revealed that optimal nimotuzumab concentrations could be achieved after a single dose regimen of 100 mg, since maximum activation of non-specific CL is reached. Doubling the dose from 50 to 100 mg increases the time in which nimotuzumab concentrations are above Kss by 150%. At higher dose levels, the increase over time is negligible (< 25%) (Figure 4). Despite the differences in the number of receptors between both compartments, the 100 mg dose saturates the non-linear elimination mechanism of nimotuzumab after administration, resulting in a parallel decrease in concentrations.

Among the most relevant limitations of the current work, the limited number of subjects included in the study (*n* = 20) and the lack of information regarding pathology, such as proteinuric condition and EGFR expression, should be highlighted, which impeded the assessment of any covariate effect on the PK parameters to partially explain the large IIV on R_totp_ and K_out_. It has been reported that several factors can influence on the PK of mAbs, for instance expression and turnover rate of targets, rate of endocytosis of mAb-target complex, mAb-neonatal Fc receptor (FcRn) interaction, immunogenicity and disease-related factors [32,41]. In the current study, immunogenicity was discarded because the human antimurine antibody response did not alter the clearance of nimotuzumab [13]. Time-dependency effects on the PK processes were not assessed since no information of multiple-dose regimens was available. Furthermore, due to the study design, it is important to note that neither the ligand nor drug–ligand complex were collected, which limited the characterization of the non-linear PK of nimotuzumab. In addition, prospective analyses are encouraged to explore the role of the mediator mechanism on the non-specific CL of nimotuzumab proposed in this article.

## 5. Conclusions

In conclusion, the developed model is a first attempt to quantitatively describe the PK and its effect of increased clearance of nimotuzumab in ADPKD. The model suggests an inverse correlation between the ratio of nimotuzumab and mediator concentration to the dose level and was able to detect that the maximum effective dose in ADPKD subjects is 100 mg. The developed model may be used in future drug development programs of nimotuzumab, guiding the understanding of the PK behavior and dose finding of nimotuzumab in other disease areas.

## Figures and Tables

**Table 1:** Characteristics of patients included in the population PK (pharmacokinetics) analysis (*n* = 20).

Characteristic		Median	Mean	Standard Deviation
Age (years)		42	39	11
Body weight (Kg)		65.7	66.98	14.69
Height (cm)		163.5	163.60	8.99
Body surface area (m^2^)		1.7	1.72	0.21
TKV (mL)	Men	678.85	822.18	486.22
	Female	846.55	924.14	404.27
TCV (mL)		310.3	339.93	201.19
Serum creatinine (mg/dL)		0.72	0.77	0.14
CrCL (mL/min/1.73 m^2^)		105.7	103.43	22.63
		*n*	%	
Race	Caucasian	15	75	
	Afro-American	1	5	
	Other	4	20	
Gender	Female	14	70	
	Male	6	30	

CrCL, creatinine clearance; TKV, total kidney volume; TCV, total cyst volume.

**Table 2:** Final parameter estimates of the population pharmacokinetic model and bootstrap analysis.

			Final PK Model	Bootstrap Analysis (*n* = 500)			
	Parameter	Units	Value	Median	RSE [%]	2.5th	97.5th
Fixed-effects	CL	[L/h]	9.64 × 10^−3^	1.00 × 10^−2^	18	6.24 × 10^−3^	1.26 × 10^−2^
	V_1_	[L]	2.63	2.65	6	2.47	2.84
	V_1_ change (D = 50 mg)	[%]	53	56	14	43	69
	Q	[L/h]	2.88 × 10^−2^	2.09 × 10^−2^	34	8.38 × 10^−3^	3.42 × 10^−2^
	V_2_	[L]	9.92 × 10^−3^	9.52 × 10^−3^	47	4.61 × 10^−3^	2.60 × 10^−2^
	K_ss_	[mg/L]	15.5	16.42	50	7.85	44.68
	k_int_	[h-1]	4.94 × 10^−3^	4.94 × 10^−3^	39	1.46 × 10^−3^	9.45 × 10^−3^
	R_tot_	[mg/L]	1.05 × 10^−2^	1.15 × 10^−2^	58	5.29 × 10^−3^	3.32 × 10^−2^
	R_totp_	[mg/L]	956	891	82	142	3481
	K_out_	[h-1]	1.33 × 10^−2^	1.36 × 10^−2^	48	5.66 × 10^−3^	3.18 × 10^−2^
	S_50_	[mg/L]	8.57	7.74	23	4.70	11.07
	S_max_		3.18	2.90	29	1.99	5.46
Inter-individual variability	R_totp_	[%]	135 (14)	158	107	65	287
	K_out_	[%]	197 (21)	226	71	131	413
Residual error	Additive	[%]	48 (4)	46	8	41	54

Eta- and Eps-shrinkage values are reported within parenthesis. RSE: relative standard error. Vss, total distribution volume at steady state: V1 + V2 = 2.64 L. CL, non-specific clearance; Q, inter-compartmental clearance; V_1_, apparent volume of distribution of the central compartment; V_2_, apparent volume of distribution of the peripheral compartment; K_SS_, steady-state rate constant; k_int_, internalization rate for nimotuzumab-EGFR; R_TOT_, concentration of free target in central compartment; R_TOTP_, concentration of free target in tissue compartment; k_out_, rate constant for the first-order degradation of the mediator; S_max_, maximal effect of the stimulation; S_50_,concentration of free nimotuzumab in the central compartment that achieves the half of S_max._
